# Supplementary material for: Within- and across-frequency temporal processing and speech perception in cochlear implant users
Source: PLoS One. 2022 Oct 13;17(10):e0275772. doi: 10.1371/journal.pone.0275772 (PMC9560480; doi:10.1371/journal.pone.0275772)
Supplement: S1 Table — (DOCX) [file pone.0275772.s001.docx]

**S1 Table. NH and CI group mean GDTs and speech perception performance**

| **Behavioral Measures** | **NH Group** | | |  | **CI Group** | | |
| --- | --- | --- | --- | --- | --- | --- | --- |
|  | **Mean ± SD** | **Minimum** | **Maximum** |  | **Mean ± SD** | **Minimum** | **Maximum** |
| CNC-Phoneme (%) | 99.7 ± 0.4 | 98.7 | 100.0 |  | 84.8 ± 9.3 | 62.7 | 95.0 |
| CNC-Word (%) | 99.1 ± 1.0 | 97.0 | 100.0 |  | 70.5 ± 12.8 | 43.0 | 85.0 |
| AzBio-Quiet (%) | 99.5 ± 0.6 | 98.3 | 100.0 |  | 88.5 ± 8.9 | 73.9 | 99.6 |
| AzBio-Noise* (%) | 99.2 ± 0.8 | 97.5 | 100.0 |  | 65.5 ± 19.2 | 38.1 | 94.3 |
| SNR-50 (dB) | -0.7 ± 1.2 | -3.0 | 1.8 |  | 8.2 ± 3.7 | 2.8 | 13.3 |
| GDT_within_ (ms) | 2.0 ± 0.0 | 2.0 | 2.0 |  | 9.6 ± 16.4 | 2.0 | 51.7 |
| GDT_across_ (ms) | 58.8 ± 38.3 | 14.6 | 120.0 |  | 82.4 ± 30.7 | 25.0 | 120.0 |
| *Note.* SNR-50 = Signal-to-Noise Ratio required for 50% correct; GDT_Within_ = Within-Frequency Gap Detection Threshold; GDT_across_ = Across-Frequency Gap Detection Threshold; * AzBio-Noise was only completed for 14 CI ears due to equipment issues. All other tests include data from 15 NH and 15 CI ears. | | | | | | | |
