# Supplementary material for: Within- and across-frequency temporal processing and speech perception in cochlear implant users
Source: PLoS One. 2022 Oct 13;17(10):e0275772. doi: 10.1371/journal.pone.0275772 (PMC9560480; doi:10.1371/journal.pone.0275772)
Supplement: S2 Table — (DOCX) [file pone.0275772.s002.docx]

**S2 Table. Behavioral mixed effect model analysis (*p-*values, F-test, degrees of freedom displayed).**

|  | **Group** | **Test Ear** | **Age at Test** |
| --- | --- | --- | --- |
| **Speech Perception** |  |  |  |
| CNC-Phoneme | **< 0.001** | 0.359 | 0.779 |
| F (DF = 5 to 12) | **26.955** | 1.012 | 0.083 |
| CNC-Word | ***< 0.001*** | 0.531 | 0.902 |
| F (DF = 3 to 8) | ***54.280*** | 0.483 | 0.017 |
| AzBio-Quiet | ***< 0.001*** | 0.850 | 0.749 |
| F (DF = 8 to 12) | ***20.008*** | 0.037 | 0.110 |
| AzBio-Noise | ***< 0.001*** | 0.279 | 0.280 |
| F (DF = 11 to 15) | ***42.645*** | 1.260 | 1.288 |
| SNR-50 | ***< 0.001*** | 0.438 | 0.116 |
| F (DF = 14 to 17) | ***72.136*** | 0.630 | 2.816 |
| **Gap Detection** |  |  |  |
| GDT_within_ | 0.158 | 0.381 | 0.152 |
| F (DF = 10 to 19) | 2.157 | 0.837 | 2.239 |
| GDT_across_ | 0.072 | 0.094 | ***< 0.001*** |
| F (DF = 7 to 18) | 3.646 | 3.732 | ***18.331*** |
| *Note.* SNR-50 = Signal-to-Noise Ratio required for 50% correct; GDT_within_ = Within-Frequency Gap Detection Threshold; GDT_across_ = Across-Frequency Gap Detection Threshold; Bold italics indicate significant p-values. | | | |
