# Supplementary material for: Within- and across-frequency temporal processing and speech perception in cochlear implant users
Source: PLoS One. 2022 Oct 13;17(10):e0275772. doi: 10.1371/journal.pone.0275772 (PMC9560480; doi:10.1371/journal.pone.0275772)
Supplement: S3 Table — (DOCX) [file pone.0275772.s003.docx]

**S3 Table. Within and across-frequency post-gap CAEP mixed effect model analysis (*p-*values, F-test, degrees of freedom displayed).**

|  | **Group** | **Condition** | **Test Ear** | **Age at Test** |
| --- | --- | --- | --- | --- |
| **Within- Frequency CAEP Measures** | | | | |
| N1-P2 Amplitude | 0.154 | 0.199 | 0.181 | ***0.041*** |
| F (DF = 19 to 74) | 2.187 | 1.662 | 1.823 | ***4.802*** |
| N1 Latency | 0.160 | 0.530 | 0.088 | 0.876 |
| F (DF = 18 to 66) | 2.152 | 0.642 | 3.001 | 0.025 |
| P2 Latency | 0.655 | 0.132 | 0.175 | 0.810 |
| F (DF = 18 to 65) | 0.206 | 2.098 | 1.881 | 0.059 |
| **Across-Frequency CAEP Measures** | | | | |
| N1-P2 Amplitude | 0.961 | 0.163 | 0.248 | 0.754 |
| F (DF = 18 to 105) | 0.003 | 1.745 | 1.350 | 0.101 |
| N1 Latency | ***0.017*** | 0.139 | ***0.012*** | ***0.010*** |
| F (DF = 14 to 95) | ***7.297*** | 1.878 | ***6.502*** | ***9.560*** |
| P2 Latency | 0.710 | 0.740 | 0.438 | ***0.003*** |
| F (DF = 15 to 108) | 0.143 | 0.418 | 0.606 | ***12.171*** |
| *Note.* Bold italics indicate significant p-values. | | | | |
