# Supplementary material for: Within- and across-frequency temporal processing and speech perception in cochlear implant users
Source: PLoS One. 2022 Oct 13;17(10):e0275772. doi: 10.1371/journal.pone.0275772 (PMC9560480; doi:10.1371/journal.pone.0275772)
Supplement: Attachment — Submitted filename: Response to Reviewers.docx [file pone.0275772.s004.docx]

Dear Dr. Prashanth Prabhu,

We greatly appreciate the thorough review of this manuscript. Together with my co-authors, I am submitting the revisions after fully considering the changes that were suggested. We believe that the manuscript is substantially improved and addresses all the comments outlined below. Our responses are shown below the reviewer’s comments in red text.

Journal Requirements:

When submitting your revision, we need you to address these additional requirements.

1. Please ensure that your manuscript meets PLOS ONE's style requirements, including those for file naming. The PLOS ONE style templates can be found at 
<https://journals.plos.org/plosone/s/file?id=wjVg/PLOSOne_formatting_sample_main_body.pdf> and 
<https://journals.plos.org/plosone/s/file?id=ba62/PLOSOne_formatting_sample_title_authors_affiliations.pdf>

Thank you for providing the links for proper formatting. I have updated the paper with PLOS ONE’s style requirements.

2. We note that the grant information you provided in the ‘Funding Information’ and ‘Financial Disclosure’ sections do not match.

When you resubmit, please ensure that you provide the correct grant numbers for the awards you received for your study in the ‘Funding Information’ section.

See response to question 3.

3. Thank you for stating the following in the Funding Section of your manuscript:

"This research was supported by the NIH NIDCD R15 DC011004 (FZ) and the University of Cincinnati Research Council (CMB)."

We note that you have provided funding information that is not currently declared in your Funding Statement. However, funding information should not appear in the Acknowledgments section or other areas of your manuscript. We will only publish funding information present in the Funding Statement section of the online submission form. 
Please remove any funding-related text from the manuscript and let us know how you would like to update your Funding Statement. Currently, your Funding Statement reads as follows:

"The funders had no role in study design, data collection and analysis, decision to publish, or preparation of the manuscript."

Please include your amended statements within your cover letter; we will change the online submission form on your behalf.

Thank you for updating the funding statement in the online submission form on my behalf. Below is the amended statement.

“This research was supported by the NIH NIDCD R15 DC011004 (Fawen Zhang) and the University of Cincinnati Research Council (Chelsea Blankenship). The funders had no role in study design, data collection and analysis, decision to publish, or preparation of the manuscript.”

We have also removed funding information from the manuscript as directed.

4. Please review your reference list to ensure that it is complete and correct. If you have cited papers that have been retracted, please include the rationale for doing so in the manuscript text, or remove these references and replace them with relevant current references. Any changes to the reference list should be mentioned in the rebuttal letter that accompanies your revised manuscript. If you need to cite a retracted article, indicate the article’s retracted status in the References list and also include a citation and full reference for the retraction notice.

We have reviewed the reference list and have verified that it is complete and correct. I have also downloaded the PLoS Reference Style for EndNote and updated the style.

[Note: HTML markup is below. Please do not edit.]

Reviewers' comments:

Reviewer's Responses to Questions

**Comments to the Author**

1. Is the manuscript technically sound, and do the data support the conclusions?

The manuscript must describe a technically sound piece of scientific research with data that supports the conclusions. Experiments must have been conducted rigorously, with appropriate controls, replication, and sample sizes. The conclusions must be drawn appropriately based on the data presented.

Reviewer #1: Partly

Reviewer #2: Yes

2. Has the statistical analysis been performed appropriately and rigorously?

Reviewer #1: Yes

Reviewer #2: I Don't Know

3. Have the authors made all data underlying the findings in their manuscript fully available?

The [PLOS Data policy](http://www.plosone.org/static/policies.action#sharing) requires authors to make all data underlying the findings described in their manuscript fully available without restriction, with rare exception (please refer to the Data Availability Statement in the manuscript PDF file). The data should be provided as part of the manuscript or its supporting information, or deposited to a public repository. For example, in addition to summary statistics, the data points behind means, medians and variance measures should be available. If there are restrictions on publicly sharing data—e.g. participant privacy or use of data from a third party—those must be specified.

Reviewer #1: Yes

Reviewer #2: Yes

4. Is the manuscript presented in an intelligible fashion and written in standard English?

PLOS ONE does not copyedit accepted manuscripts, so the language in submitted articles must be clear, correct, and unambiguous. Any typographical or grammatical errors should be corrected at revision, so please note any specific errors here.

Reviewer #1: Yes

Reviewer #2: Yes

5. Review Comments to the Author

Please use the space provided to explain your answers to the questions above. You may also include additional comments for the author, including concerns about dual publication, research ethics, or publication ethics. (Please upload your review as an attachment if it exceeds 20,000 characters)

Reviewer #1: The authors made an attempt to study the behavioral and electrophysiological GDT in normal hearing and CI users and compared it speech perception in quiet and noise. The design of the study is appreciable. However, following queries needs to be addressed before considering for publication.
1. Line 116: Ambiguous sentence

We have revised the ambiguous sentence to the following:

“The authors of the present study reported that CI recipients with smaller GDT_within_ had better speech perception scores for words in quiet and sentences in noise [18].”

2. Line 193: The reason for taking 2kHz and 1 kHz in particular was not justified.

We added the following justification for using the 1 and 2 kHz markers. “Furthermore, the marker frequencies (1 and 2 kHz) were selected to facilitate comparison to studies that used a similar paradigm in adults with normal hearing or minimal hearing loss [32, 33].”

3. Line 223: The authors used different transducers for NH and CI for behavioral GDT which was not justified.

Different transducers were used due to the software needed to run the behavioral GDT. Behavioral GDT were measured using APEX software that we were unable to route through the audiometer.

4. Line 240-241: The authors need to clarify how was the reference (no gap) stimuli constructed for GDT across for 2AFC.

I have added the following to clarify “The no gap stimuli was a continuous 1 or 2 kHz tone that was constructed similarly to the target stimuli (e.g., cos^2^ window and also included a 10 ms rise-fall time).”

5. The soundcard details of the personal computer is not provided. Further, were the stimuli routed through audiometer? If so, was the details of the audiometer to be given.

No the stimuli were not routed through the audiometer. The sound card details have been added to the manuscript.

6. Pictorial representation of the stimuli for GDT needs to be provided for better understanding.

This has been added as Fig 1 and is under the stimuli section.

7. Test-retest reliability is not checked for this study. Authors need to clarify.

That is correct, we did not specifically examine test-retest reliability within this study. However previous studies have shown high test-retest reliability for gap detection tasks. Additionally for the speech perception tests, we measured two lists per test for each participant. Within each test, individual participant scores were very consistent between lists.

8. The mean thresholds of CI users at 4 kHz and 6 kHz are higher. This is unusual as most of the time the thresholds are around 25-30 dB even for high frequencies in the CI users.

We agree with the reviewer’s observation that the CI thresholds at 4 and 6 kHz are slightly higher than expected. All the CI participants were recruited from a large CI clinic, irrespective of performance, where they were regularly seen for programming appointments and equipment checks. Prior to testing we checked every participants’ microphone covers to see if they needed changed and we instructed them to set their speech processor to their everyday settings. For some individuals with increased durations of auditory deprivation, it can be more difficult to attain high-frequency audibility post-implantation. The participants in the current study had auditory deprivation that ranged from 10 to > 50 yrs, which might be a factor relating to the higher aided audiometric thresholds at 4 and 6 kHz.

9. The discussion for CAEP vs Speech performance is inadequate.

We have expanded the discussion of the CAEP vs speech perception to include more information about the significant correlations and the relationship to the literature.

Reviewer #2: Overall the methods of the study were clear and the objectives were accomplished. The literature review was extensive and covered the topic thoroughly. The individual discussion sections on the individual effects seem to be orphaned from the final concluding remarks and conjecture (the traditional discussion section) and read more as an extension of the results section with a comparison to literature more than providing rationale in literature and then explaining the significance of the correlation. A reorganization of text is suggested for the discussion section, however, all of the data and conjecture is there. No additional information is needed.

Based on the reviewer’s comments above, I have modified the discussion and conclusion section to tie everything together better and to provide more of a rationale for the results rather than just comparing them to the literature.

There is a great deal of supplementary material that may be better served as 1-2 reference lines in the results section to include the mean/std of the CEAP data.

This change has been made as suggested and two tables (within and across mean/sd CAEP values) have been moved from supplementary material to the results section.

Lastly, there were a lot of various statistical analyses performed. While not my expertise, a direct comparison of individual effects using the mixed effects model could be better explained. The study was well written and clear and of significance to the field of cochlear implants and speech science.

I have added additional information to the statistical analysis section to explain why linear mixed effect models were used for analysis. Additionally, Jareen Meinzen-Derr, co-author and Director of the Biostatistics, Epidemiology, and Research Design Program at Cincinnati Children’s Hospital was consulted on all analysis. She helped design the mixed effect models, verify the analysis steps were appropriate and provide guidance on interpretation of results.

Minor Comments:
Line 94: Do you mean, “markers”?

Yes, thank you for catching the typo, it has been corrected.

Line 102: Typically, “stimulation mode” refers to the nature of the grounding of the electrical circuit or the type of the pulse (monopolar, tripolar, etc.). Perhaps use “experimental modalities” or “presentation modalities”

Thank you for the suggestion, we have changed “stimulation mode” to presentation modalities”

Line 104: Suggest using naturalistic audio instead of “daily life sounds”

Need to address because I don’t like the term naturalistic audio

I have modified this section for the comment below and “daily life sounds” is no longer in the manuscript.

Lines 103-105: Does this sentence mean to say that the resulting response of the stimuli provides a better response with the clinical processor and microphone than directly stimulating the intracochlear array? Is there any literature, or have the authors performed this task, regarding the comparison of the same task across different presentation approaches? Ideally these experiments take place inside a sound booth, in a controlled manner which would leave the researcher to assume the input from the microphones and the input sent to the clinical processor directly would be the same.

No, I was not trying to indicate that there might be a better response when sound is processed though the clinical speech processor than with direct electrical stimulation. I was trying to convey the idea that using acoustic stimuli presented through a speaker is more representative of how CI recipients hear during a typical day and may provide a more accurate measure of temporal processing.

I have modified the text to clarify.

“In hearing-impaired listeners using a cochlear implant (CI), a prosthetic device that converts sounds into electrical pulses that directly stimulate the auditory nerve. GDTs can be evaluated using different presentation modalities including acoustic stimulation and direct electrical stimulation (by passes the CI speech processor). Acoustic GDTs reflect the temporal processing impairments within the auditory system and signal distortion associated with the speech processing strategy and individual map parameters (stimulation rate, maxima, preprocessing strategies). Therefore, acoustic GDTs are expected to be poorer than those measured with direct electrical stimulation and are more representative of temporal processing abilities in everyday situations. Examining CI users’ GDTs using acoustic stimulation may provide valuable information on why the clinical outcomes of cochlear implantation are highly variable across individuals.”

Line 112: Check the in-text reference to [20] to ensure it matches the other references

I have fixed the in-text reference style throughout the entire manuscript.

Line 116, Lines 428-429: Preferential use of first person pronoun, suggest stating “the authors of the present study reported that…”

Change made as suggested.

Line 121: “increases”

Change made as suggested.

Line 145: Check the in-text reference style

I have fixed the in-text reference style throughout the entire manuscript.

Line 185: “marker”

Thank you for checking this error, this was corrected here and 4 other instances.

Line 212: Check the in-text reference style

I have fixed the in-text reference style throughout the entire manuscript.

Line273: “Behavioral data” can mean a wide variety of thing, typically used for psychology-based experiments. Perhaps use “subjective data” or “performance” – the authors use “speech perception” which would also be appropriate

Changed to speech perception and gap detection data

Line 302: “makers”

Thank you for checking this error, this was corrected here and 4 other instances.

Line 332: Remove the “a” before “much higher”

Removed as suggested.

Figure 2: Typically this is useful for validation purposes such as dissertations, but may not be needed here and could save space in the journal (to potentially include some of the supplemental data here instead).

Removed as suggested and instead moved the group mean tables to the manuscript.

Line 396: “Bonferroni corrections were applied…”

Change made as suggested.

Line 397 and Line 404: “…therefore a p-value of 0.01 was…”

Change made as suggested.

Line 403-404: Are the authors using the post-hoc, multiple comparisons tests using Bonferroni corrections? These explicit comparison groups using MC analysis should be more clear in the methods section

We have updated the statistical analysis section to include more detailed information for the correlation analysis using Bonferroni corrections.

Lines 423-424: The difficult of the task between CI and NH users should be equivalent such that there are no ceiling level effects for either group, especially for NH users. In literature, many CI studies which use NH data will lower the SNR for the NH group while keeping the CI SNR somewhat higher. While the tests cannot be completed again, a stronger correlation could be had when this factor is controlled between groups.

We agree with this observation and have added this as a limitation to the study.

Line 425-426: Did the authors consider the performance level of the CI group? For example, splitting the data into groups based on their performance level in quiet with their clinical processor. This type of analysis could also provide insight on the large variability seen with CI studies. Although the study was age-matched, it could be more impactful if the study was also matched based on performance. For CI users, this is incredibly difficult to do, but would be a worth-while comparison at the end of the day.

Yes we did consider grouping the CI recipients into good and poor performers based on their behavioral scores, however due to the small sample size this analysis was not able to be performed.

Line 429: A period is needed after the (18, 46,62) reference

Change made as suggested.

Line 429, Line 452-453: “In a previous study by the authors,… a mean CNC-Word score of … was reported…”

Change made as suggested.

Line 452: Include the take-away from the previous studies on GDTwithin or remove the sentence. This is covered in the introduction (or if new literature, should be added).

The authors feel like this is not needed and have removed it as the reviewer suggested.

Line 454: Remove “a” before noise, or include stimuli after “a”

Change made as suggested.

Lines 450-464: The correlation of the GDTwithin and speech perception of the present study is not clear here or how it relates to the mentioned literature. Please elaborate. A better comparison is needed.

We agree with the reviewer and have removed this information from the behavioral GDT discussion section. The authors have moved this information to the Correlation analysis section (GDT_within_ vs. speech perception; GDT_across_ vs. speech perception).

Line 452: “shorter”

Change made as suggested.

Line 465: “The difference between NH and CI users in GDTacross scores, regardless of the wide variability was not found to be significant.”

Change made as suggested.

Lines 489-495: include with previous paragraph

Change made as suggested.

Line 536: “Markers”

Change made as suggested.
