# Supplementary material for: Within- and across-frequency temporal processing and speech perception in cochlear implant users
Source: PLoS One. 2022 Oct 13;17(10):e0275772. doi: 10.1371/journal.pone.0275772 (PMC9560480; doi:10.1371/journal.pone.0275772)
Supplement: Attachment — Submitted filename: Response to Reviewers.docx [file pone.0275772.s005.docx]

Dear Dr. Prashanth Prabhu,

We greatly appreciate the thorough review of the revised manuscript. Together with my co-authors, I am submitting the revisions after fully considering the changes that were suggested. We believe that the manuscript is substantially improved and addresses all the comments outlined below. Our responses are shown below the reviewer’s comments in red text.

PONE-D-22-13719R1

Within- and across-frequency temporal processing and speech perception in cochlear implant users

PLOS ONE

Dear Dr. Blankenship,

Thank you for submitting your manuscript to PLOS ONE. After careful consideration, we feel that it has merit but does not fully meet PLOS ONE’s publication criteria as it currently stands. Therefore, we invite you to submit a revised version of the manuscript that addresses the points raised during the review process.

Please submit your revised manuscript by Oct 28 2022 11:59PM. If you will need more time than this to complete your revisions, please reply to this message or contact the journal office at [plosone@plos.org](mailto:plosone@plos.org). When you're ready to submit your revision, log on to <https://www.editorialmanager.com/pone/> and select the 'Submissions Needing Revision' folder to locate your manuscript file.

Please include the following items when submitting your revised manuscript:

- A rebuttal letter that responds to each point raised by the academic editor and reviewer(s). You should upload this letter as a separate file labeled 'Response to Reviewers'.
- A marked-up copy of your manuscript that highlights changes made to the original version. You should upload this as a separate file labeled 'Revised Manuscript with Track Changes'.
- An unmarked version of your revised paper without tracked changes. You should upload this as a separate file labeled 'Manuscript'.

If you would like to make changes to your financial disclosure, please include your updated statement in your cover letter. Guidelines for resubmitting your figure files are available below the reviewer comments at the end of this letter.

If applicable, we recommend that you deposit your laboratory protocols in protocols.io to enhance the reproducibility of your results. Protocols.io assigns your protocol its own identifier (DOI) so that it can be cited independently in the future. For instructions see: <https://journals.plos.org/plosone/s/submission-guidelines#loc-laboratory-protocols>. Additionally, PLOS ONE offers an option for publishing peer-reviewed Lab Protocol articles, which describe protocols hosted on protocols.io. Read more information on sharing protocols at <https://plos.org/protocols?utm_medium=editorial-email&utm_source=authorletters&utm_campaign=protocols>.

We look forward to receiving your revised manuscript.

Kind regards,

Prashanth Prabhu
Academic Editor
PLOS ONE

Journal Requirements:

Please review your reference list to ensure that it is complete and correct. If you have cited papers that have been retracted, please include the rationale for doing so in the manuscript text, or remove these references and replace them with relevant current references. Any changes to the reference list should be mentioned in the rebuttal letter that accompanies your revised manuscript. If you need to cite a retracted article, indicate the article’s retracted status in the References list and also include a citation and full reference for the retraction notice.

I have reviewed the reference list to ensure that it is complete and correct.

Additional Editor Comments:

The authors have incorporated most of the suggestions provided by the reviewers. There are few minor concerns still which should be addressed before its considered for publication. 

[Note: HTML markup is below. Please do not edit.]

Reviewers' comments:

Reviewer's Responses to Questions

**Comments to the Author**

1. If the authors have adequately addressed your comments raised in a previous round of review and you feel that this manuscript is now acceptable for publication, you may indicate that here to bypass the “Comments to the Author” section, enter your conflict of interest statement in the “Confidential to Editor” section, and submit your "Accept" recommendation.

Reviewer #1: All comments have been addressed

Reviewer #3: (No Response)

2. Is the manuscript technically sound, and do the data support the conclusions?

The manuscript must describe a technically sound piece of scientific research with data that supports the conclusions. Experiments must have been conducted rigorously, with appropriate controls, replication, and sample sizes. The conclusions must be drawn appropriately based on the data presented.

Reviewer #1: Yes

Reviewer #3: Yes

3. Has the statistical analysis been performed appropriately and rigorously?

Reviewer #1: Yes

Reviewer #3: Yes

4. Have the authors made all data underlying the findings in their manuscript fully available?

The [PLOS Data policy](http://www.plosone.org/static/policies.action#sharing) requires authors to make all data underlying the findings described in their manuscript fully available without restriction, with rare exception (please refer to the Data Availability Statement in the manuscript PDF file). The data should be provided as part of the manuscript or its supporting information, or deposited to a public repository. For example, in addition to summary statistics, the data points behind means, medians and variance measures should be available. If there are restrictions on publicly sharing data—e.g. participant privacy or use of data from a third party—those must be specified.

Reviewer #1: Yes

Reviewer #3: Yes

5. Is the manuscript presented in an intelligible fashion and written in standard English?

PLOS ONE does not copyedit accepted manuscripts, so the language in submitted articles must be clear, correct, and unambiguous. Any typographical or grammatical errors should be corrected at revision, so please note any specific errors here.

Reviewer #1: Yes

Reviewer #3: Yes

6. Review Comments to the Author

Please use the space provided to explain your answers to the questions above. You may also include additional comments for the author, including concerns about dual publication, research ethics, or publication ethics. (Please upload your review as an attachment if it exceeds 20,000 characters)

Reviewer #1: The discussion for CAEP and speech perception needs to be revised further. The discussion should explain the presence of smaller N1-P2 amplitude and longer N1 latency and its significance to poor speech perception.

We have added information about the relationship between CAEP and speech perception performances to lines 671-700.

Reviewer #3: General Comments
• Well thought research experiment.
• As rightly mentioned in the limitations, considerations of the smaller gaps and rise time of the GDT stimulus would have further strengthen the methods.
• Explanations provided by the authors are fine but already existing and not conclusive (It cannot be either with the study undertaken). However, the need is to define the actual processes responsible for these phenomena.

Introduction
1. Line 148 to 156: Relation of CAEP peak’s amplitude and latency with the change in GDT – stimuli – paradigm should be mentioned more clearly in the Introduction (in NH population, especially) to provide a better idea in the relationship to readers naïve to this topic.

We have added more information about the effect of gap duration on CAEP amplitude and latency to lines 140-169.

Methods
2. Most of the stimulus presentation is via the speaker. Information on the make of the speaker to be added.

We have added information about the speakers to lines 247 and 261.

3. Line 242 to 245: What was the need of occluding the contralateral ear while estimating Speech Perception in CI patients? Just switching off the processor of that side would have done it.

Yes for bilaterally implanted CI recipients we removed the contralateral speech processor. However some CI recipients were unilaterally implanted. Therefore to keep it consistent across all participants we removed contralateral devices if applicable and then occluded the contralateral ear during testing.

This information is already contained on Lines 235-238: “For NH individuals and unilateral CI users, testing was completed with the contralateral ear occluded with an E-A-R disposable foam ear plug. For bilateral CI users, testing was completed with the contralateral speech processor removed.”

4. GDT involves the processing of the shortest gap which is more dependent on cortical processing. Therefore, to me, having 2 ears of the same person in the study is not right. Because you will be tapping the same cortex. Justify, please.

I agree with the reviewer that the responses from the left and right ear of the same participant are not going to be completely independent of each other. This is why we included participant ID as a random effect in the model to account for similar variabilities in ears “clustered” around an individual. Furthermore, while I agree that cortical processing is important, there are other sub-cortical nuclei within the central auditory nervous system are critical for temporal processing. For example, the inferior colliculus contain neurons that are sensitive to stimulus duration, essential to the perceptual ability to detect temporal changes in the stimulus, such as gap detection or amplitude modulation [1]. Animal studies of single neurons within the mouse inferior colliculus have demonstrated electrophysiological GDTs that are within 1 to 2 ms of behavioral GDTs [2]. So there are ear specific aspects of temporal processing that are critical to gap detection thresholds and CAEPs.

This is also apparent in the raw data. There are some participants with behavioral GDTs that have very similar thresholds for the left and right ear while others show differences in thresholds up to > 20 ms. The same holds true for the CAEP amplitude and latency values. For example, some participants have left and right N1-P2 amplitude values that are very similar (0.98 vs 1.03 µV), others display large differences in amplitude (1.29 vs 3.64 µV). So the results across ears are not necessarily identical and we have taken steps to factor the correlation in responses into our analysis.

Results
5. Sample of the study is very small, therefore the inclusion of the analysis of the effect of age and ear is inappropriate.

We admit that the small sample size is a limitation of this study. Thus, we worked closely with an experienced biostatistician (Jareen Meinzen-Derr) for both study planning and statistical analyses.  Models that were constructed testing the feasibility of the inclusion of age and ear to avoid overfitting the model.  Additionally, we are aware of debates regarding sample size in neuroscience studies. Our study included 15 CI ears and 15 NH control ears among 22 participants; such a sample size is not uncommon for EEG studies involving CI users [3-5]. We strongly believe the significant findings of our work will lead to future research efforts of examining temporal processing in CI users with larger sample sizes.

Discussion
6. Line 783: Insert comma after Additionally.

Change made as suggested.

7. PLOS authors have the option to publish the peer review history of their article ([what does this mean?](https://journals.plos.org/plosone/s/editorial-and-peer-review-process#loc-peer-review-history)). If published, this will include your full peer review and any attached files.


If you choose “no”, your identity will remain anonymous but your review may still be made public.


**Do you want your identity to be public for this peer review?** For information about this choice, including consent withdrawal, please see our [Privacy Policy](https://www.plos.org/privacy-policy).

Reviewer #1: No

Reviewer #3: **Yes:** UDIT SAXENA

**References:**

1.	Faure PA, Fremouw T, Casseday JH, Covey E. Temporal masking reveals properties of sound-evoked inhibition in duration-tuned neurons of the inferior colliculus. J Neurosci. 2003;23(7):3052-65. Epub 2003/04/10. PubMed PMID: 12684492.

2.	Walton JP, Frisina RD, Ison JR, O'Neill WE. Neural correlates of behavioral gap detection in the inferior colliculus of the young CBA mouse. Journal of comparative physiology A, Sensory, neural, and behavioral physiology. 1997;181(2):161-76. Epub 1997/08/01. PubMed PMID: 9251257.

3.	Rahne T, Plontke SK, Wagner L. Mismatch negativity (MMN) objectively reflects timbre discrimination thresholds in normal-hearing listeners and cochlear implant users. Brain Res. 2014;1586:143-51. Epub 2014/08/26. doi: 10.1016/j.brainres.2014.08.045. PubMed PMID: 25152464.

4.	Wagner L, Plontke SK, Rahne T. Perception of Iterated Rippled Noise Periodicity in Cochlear Implant Users. Audiol Neurootol. 2017;22(2):104-15. Epub 2017/08/30. doi: 10.1159/000478649. PubMed PMID: 28848077.

5.	Timm L, Agrawal D, F CV, Sandmann P, Debener S, Buchner A, et al. Temporal feature perception in cochlear implant users. PLoS One. 2012;7(9):e45375. Epub 2012/10/03. doi: 10.1371/journal.pone.0045375. PubMed PMID: 23028971; PubMed Central PMCID: PMCPMC3448664.
